# Low-Temperature Fabrication of BiFeO_3_ Films on Aluminum Foils under a N_2_-Rich Atmosphere

**DOI:** 10.3390/nano14161343

**Published:** 2024-08-14

**Authors:** Jing Yan

**Affiliations:** College of Physics and Electronic Engineering, Qilu Normal University, Jinan 250200, China; jndxclyj@163.com

**Keywords:** BiFeO_3_, aluminum foil, N_2_-rich atmosphere, electrical properties

## Abstract

To be CMOS-compatible, a low preparation temperature (<500 °C) for ferroelectric films is required. In this study, BiFeO_3_ films were successfully fabricated at a low annealing temperature (<450 °C) on aluminum foils by a metal–organic decomposition process. The effect of the annealing atmosphere on the performance of BiFeO_3_ films was assessed at 440 ± 5 °C. By using a N_2_-rich atmosphere, a large remnant polarization (*P_r_*~78.1 μC/cm^2^ @ 1165.2 kV/cm), and a high rectangularity (~91.3% @ 1165.2 kV/cm) of the *P-E* loop, excellent charge-retaining ability of up to 1.0 × 10^3^ s and outstanding fatigue resistance after 1.0 × 10^9^ switching cycles could be observed. By adopting a N_2_-rich atmosphere and aluminum foil substrates, acceptable electrical properties (*P_r_*~70 μC/cm^2^ @ 1118.1 kV/cm) of the BiFeO_3_ films were achieved at the very low annealing temperature of 365 ± 5 °C. These results offer a new approach for lowering the annealing temperature for integrated ferroelectrics in high-density FeRAM applications.

## 1. Introduction

BiFeO_3_ (BFO) has been extensively investigated as a promising multiferroic material in recent years [1,2,3,4,5,6,7,8]. Studies have reported various approaches for enhancing the ferroelectric properties of BFO films, such as by introducing buffer layers, doping the isovalent or aliovalent ions, and domain engineering [9,10,11,12,13,14,15,16,17]. However, obtaining *P-E* hysteresis loops with high rectangularity in polycrystalline BFO films using chemical solution deposition methods remains a challenge, especially under low annealing temperatures (<500 °C). Most efforts have focused on improving the quality of BFO films [9,10,11,12,13,14,15,16,17,18,19]; however, few studies have focused on the bottom electrode as well as the interface between the bottom electrode and the film, even though these factors remain equally important. In general, obtaining the desired contact at the interface between the traditional substrate (such as Si) and the film may be difficult to achieve due to slight thermal deformations during the low-temperature (<500 °C) annealing treatment. Recently, Kingon et al. successfully obtained high-quality Pb(Zr_0.52_Ti_0.48_)O_3_ films directly on base metal copper foils, providing a new strategy for choosing base metal foils as the bottom electrodes [20]. As a common base metal, aluminum (Al) foil may serve as an alternative electrode for BFO ferroelectric films as Al_2_O_3_ can readily form a very dense, stable, and extremely thin (~5 nm) layer. This may effectively reduce the leakage current and lower the risk of breakdown. Furthermore, the thermal expansion coefficient of Al (23.8 × 10^−6^/°C) [21] is much higher than that of Si (3.6 × 10^−6^/°C) [22]; thus, a tight contact interface between the BFO film and the Al substrate can be expected, even at a low annealing temperature. In this work, aluminum foils were adopted as substrates for the preparation of BFO films using the metal–organic decomposition (MOD) method. High temperatures often cause serious problems, including interdiffusion, charged defects, phase decomposition, and valence fluctuations, and these issues can damage a film’s electrical properties and performance stability [23]. To enhance the performance of BFO films and to provide complementary metal oxide semiconductor (CMOS) compatibility, a low processing temperature below 500 °C was required. The effects of annealing atmospheres on the properties of BFO films on Al substrates were discussed, and it was found that a N_2_-rich atmosphere could facilitate the crystallization of BFO films and thus lower the annealing temperature. Therefore, low-temperature preparation of BFO films was attempted. By adopting a N_2_-rich atmosphere and aluminum foil substrates, adequate ferroelectric properties (*P_r_*~70 μC/cm^2^ @ 1118.1 kV/cm) were obtained at a very low annealing temperature of 365 ± 5 °C.

## 2. Materials and Methods

BFO films (~800 nm) were fabricated on mirror aluminum foils (surface roughness ~0.02 ± 0.005 μm; thickness ~0.3 mm; size ~10 mm × 10 mm) using the MOD process. The precursor solution was prepared by dissolving bismuth nitrate and iron nitrate in acetic acid and ethylene glycol according to the stoichiometric ratio (All the chemical reagents were purchased from Sinopharm Chemical Reagent, Shanhai, China). Excess 5 mol% bismuth was used to compensate for the volatilization of Bi_2_O_3_. The ratio of acetic acid and ethylene glycol was 3:1, and the solution concentration was 0.1 mol/L. The films were deposited onto the Al foils by spin-coating and then annealed layer by layer for 10 min at 365~440 ± 5 °C in N_2_-rich (BFO_N_) or O_2_-rich (BFO_O_) atmospheres (during the annealing process, N_2_ or O_2_ was introduced at a rate of 1.5 sccm). Each layer of film was deposited onto the substrate by spin-coating at 4000 rpm for 30 s. Au top electrodes were deposited onto the films using a sputtering system through a shadow mask with a diameter of 0.2 mm. Crystallographic characteristics of BFO films were analyzed by using standard X-ray diffraction (XRD) 2θ-scans in a Dmax-2500PC diffractometer (Rigaku, Tokyo, Japan) equipped with a Ni-filtered Cu-Kα radiation source (λ = 1.54184 Å). Surface morphologies were characterized using a SU-70 thermal field-emission scanning electron microscope (SEM) (Hitachi, Tokyo, Japan). The chemical bonding states of the constituent elements were analyzed using a Thermo Scientific™ K-Alpha™ (hν = 1486.6 eV) X-ray Photoelectron Spectrometer (Thermo Fisher Scientific, Waltham, MA, USA). The energy resolution and the spatial resolution of the spectrometer were 0.5 eV and 50 μm, respectively. A Precision Premium II ferroelectric tester (maximum voltage of 99.9 V, Radiant Technology, Albuquerque, NM, USA) was adopted to measure the ferroelectric properties and leakage currents. The soaking time was 500 ms during the leakage test under the unswitched linear mode.

## 3. Results and Discussion

### 3.1. Performance of BiFeO_3_ Annealed at 440 ± 5 °C under Different Atmospheres

#### 3.1.1. Microstructure Analysis

**Figure 1:**
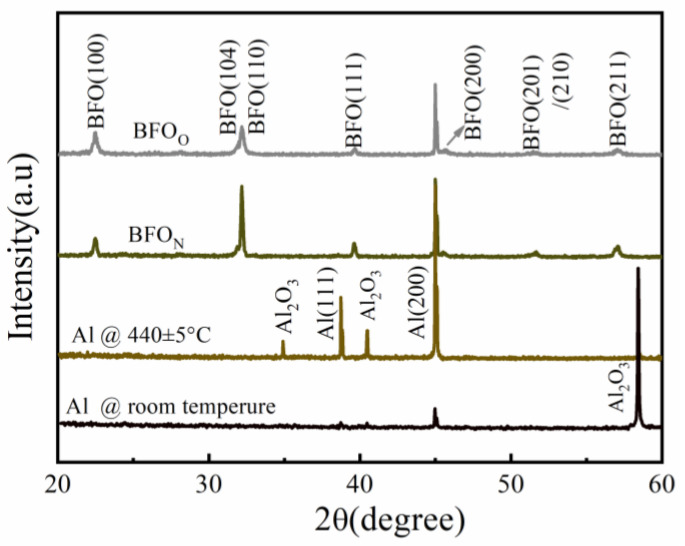
(Color online) X-ray diffraction (XRD) 2θ-scan patterns of the Al foil and BFO films deposited in N_2_-rich and O_2_-rich atmospheres at 440 ± 5 °C.

**Figure 2:**
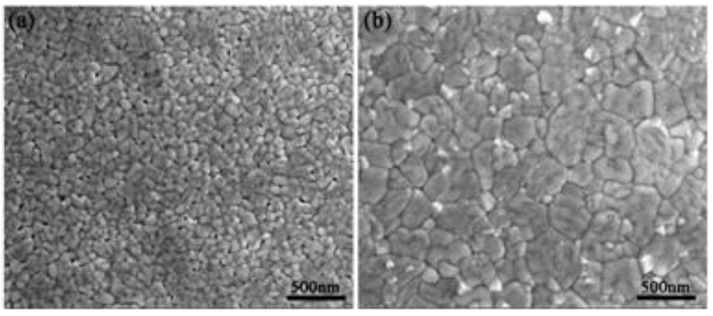
Surface SEM images for (**a**) the BFO_O_ and (**b**) BFO_N_ films at 440 ± 5 °C.

The polycrystalline perovskite structures with a bulk-like rhombohedral phase were obtained in both the BFO_N_ and BFO_O_ films at 440 ± 5 °C [23], as shown in Figure 1. No secondary phase was observed. This demonstrated that the Al foil was a suitable substrate material for the preparation of BFO films. The global average grain size calculated via the Scherrer formula for the BFO_N_ and BFO_O_ films are shown in Table 1. The grain size of the BFO_N_ film was larger than that of the BFO_O_ film. The local average grain sizes via SEM analysis (Figure 2, from a statistical analysis of 100 grains via the Nano Measurer 1.2.5 software) are also shown in Table 1. The similar results indicate that a reduced atmosphere can facilitate grain growth. The difference between the global average grain size and the local grain size may be related to the grain size nonuniformity.

Figure 2 shows the surface morphologies of the BFO films. A compact surface and larger grains were observed in the BFO_N_ films compared with the BFO_O_ films at 440 ± 5 °C, possibly due to the higher content of oxygen vacancies under a reducing atmosphere than that in an O_2_-rich atmosphere. Reports have demonstrated that oxygen vacancies can facilitate the diffusion of ions during the annealing process [24]. As shown in Figure 2b, the BFO_N_ film was mainly composed of large block-like grains, which could be ascribed to the presence of the grain merger phenomenon during the grain growth process. Notably, the observed white fine grains on the surface of the BFO_N_ film may be Bi_2_O_3_ grains, because the relatively high content of oxygen vacancies in the BFO_N_ film possibly accelerated the diffusion of Bi_2_O_3_. As a result, significantly more Bi_2_O_3_ potentially migrated from the interior to the surface. From Figure 2a, a granular surface morphology with uniform small grains were observed in the BFO_O_ film. Obvious voids were located at the grain boundaries, deteriorating the densification of the BFO_O_ film and its electrical properties.

**Figure 3:**
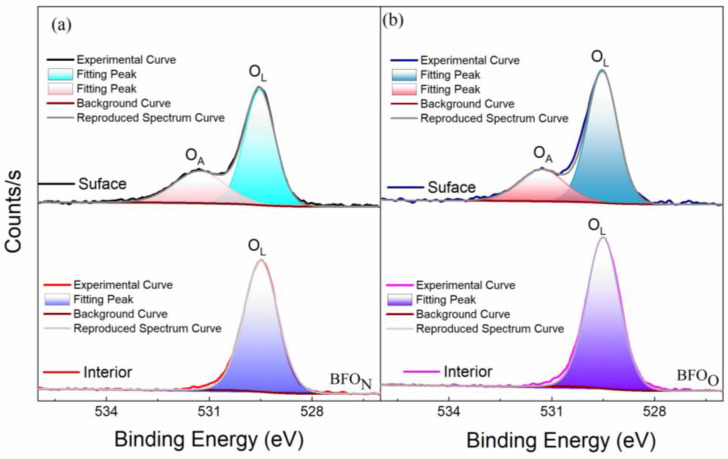
(**a**,**b**) are the XPS spectra of the O1s core level for the surface as well as the interior of the BFO_O_ and BFO_N_ thin films, respectively.

The X-ray photoelectron spectroscopy (XPS) spectrum of O1s core levels of the BFO_O_ and BFO_N_ thin films are shown in Figure 3. A strong peak at ~529.5 eV was observed for the BFO_O_ and BFO_N_ thin films, which corresponds to oxygen in the perovskite lattice (O_L_). The peak at ~531.2 eV is the surface-absorbed oxygen (O_A_) [23]. To verify this, the surface of the BFO_O_ and BFO_N_ films were etched to a thickness of ~3 nm. No peaks could be obtained at ~531.2 eV at the interior of the BFO_O_ and BFO_N_ thin films. Furthermore, no obvious oxygen vacancy peak was observed in the BFO films, which may be related to the low number of oxygen vacancies in BFO films annealing in a rapid thermal annealing furnace in air.

#### 3.1.2. Room-Temperature Electrical Properties

**Figure 4:**
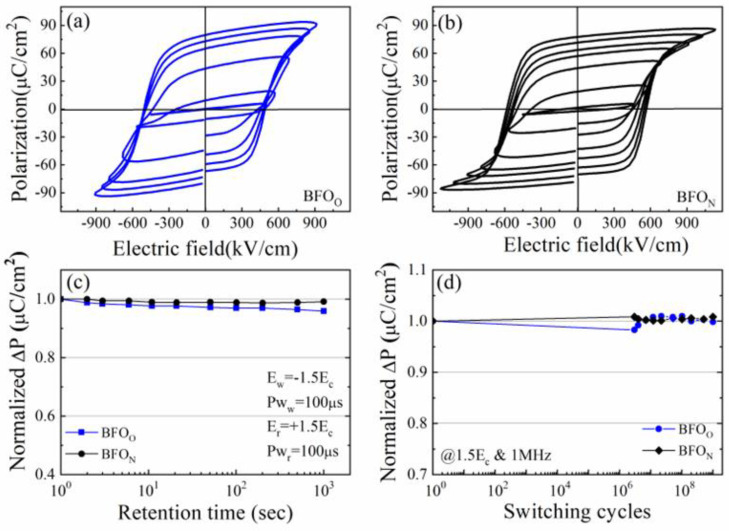
(Color online) room-temperature polarization–electric field (*P*-*E*) curves for (**a**) the BFO_O_ and (**b**) BFO_N_ films at 440 ± 5 °C. (**c**) Retention and (**d**) fatigue properties investigated for the BFO_O_ and BFO_N_ films at 440 ± 5 °C.

Figure 4 shows the electrical properties of BFO_O_ and BFO_N_ films at 440 ± 5 °C. Figure 4a,b present the typical P-E loops of the BiFeO_3_ films. When the applied electric field was sufficiently large, well-formed rectangular-shaped, saturated P-E hysteresis loops were observed in the BFO_N_ and BFO_O_ films. A large remanent polarization (P_r_~78.1 μC/cm^2^) and high rectangularity (~91.3%) of the hysteresis loop were observed in the BFO_N_ film at the applied electric field of 1165.2 kV/cm. A high remanent polarization (P_r_~79.6 μC/cm^2^) and good rectangularity (~86.8%) of the hysteresis loop were observed in the BFO_O_ film at the applied electric field of 915.4 kV/cm. Compared with the BFO_N_ film, the P-E loops of the BFO_O_ film displayed a somewhat roundish shape, indicating contributions from the leakage current induced by the voids, and more grain boundaries [25]. The normalized pulsed polarization (ΔP = P* (switched polarization) − Pˆ (nonswitched polarization), their retention time, and switching cycles are shown in Figure 4c,d. Both the BFO_N_ and BFO_O_ films exhibited good charge-retaining ability for up to 1.0 × 10^3^ s. The improved charge-retaining ability (~1% loss) of the BFO_N_ film compared with that (~4% loss) for the BFO_O_ film was attributed to the reduced domain backswitching induced by the grain boundary defects and voids in the former compared with the latter [25]. As shown in Figure 4d, good fatigue resistance of up to 1.0 × 10^9^ switching cycles was observed in both the BFO_N_ and BFO_O_ films, which could be attributed to the improved interface from using Al substrates as well as the low oxygen vacancies, as discussed in Figure 3.

**Figure 5:**
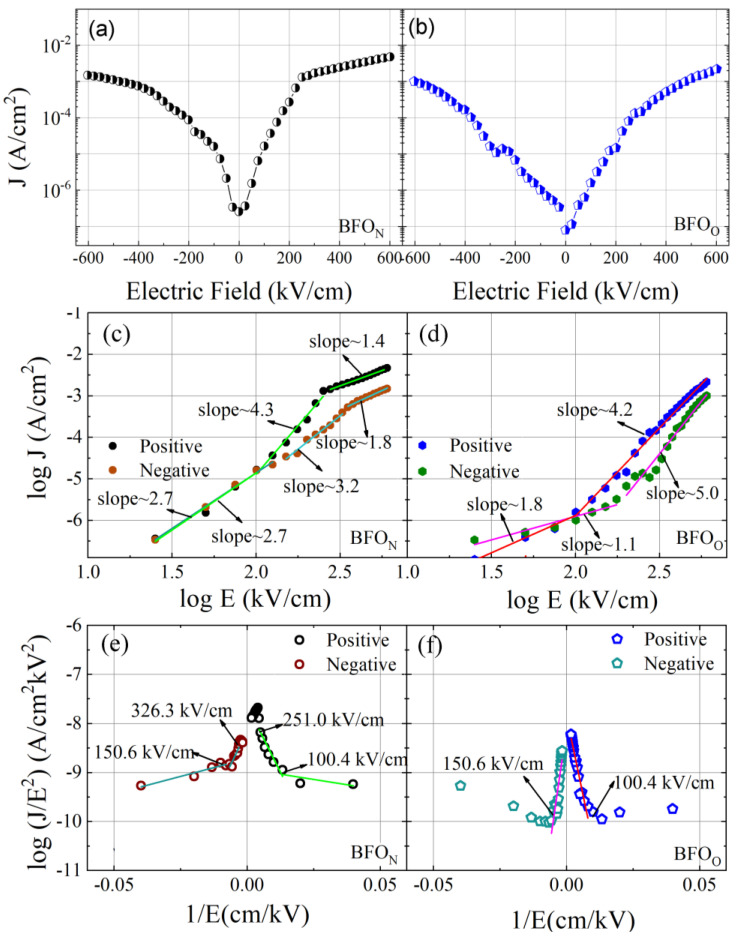
(Color online) (**a**,**b**) are the leakage current density–electric field (J–E) curves for BFO_O_ and BFO_N_ films annealed at 440 ± 5 °C, respectively. (**c**–**f**) are the curve-fitting results for the J–E curves in (**a**,**b**).

Leakage performances for the BFO_O_ and BFO_N_ films are shown in Figure 5; the soaking time was 500 ms during the leakage test under the unswitched linear mode [26]. From Figure 5a,b, we can see that the leakage current density (J) of the BFO_N_ film was slightly higher than 1-fold that of BFO_O_ film but less than 2-fold, which can be ascribed to the slightly lower oxygen vacancy concentration in the BFO_O_ film, as discussed in Figure 3. To clarify the dominant leakage mechanism involved in BFO films, linear fittings of the leakage behavior are shown in Figure 5c–f based on leakage mechanism formulas [27]. From Figure 5c,e, the Fowler–Nordheim tunneling (FN tunneling) [ln (J/E^2^)∝1/E] mechanism is induced by the interface electrons activated into the conduction band by the tunneling involved in the BFO_N_ film under the positive electric field (<251.0 kV/cm) [28,29]. The FN tunneling mechanism observed at the low electric field may be related to the good crystallization of the BFO_N_ film. The Ohmic conduction mechanism (log J∝log E) can be observed at a high electric field (>251.0 kV/cm), which should be related to the free oxygen vacancies in the film under the positive electric field. With an increasing electric field, the electrons transitioning from the interface to the body may form defect complexes with oxygen vacancies. The formation of defect complexes suppresses the electron transition from the interface to the conduction band and changes the leakage mechanism [30]. In a negative electric field, the FN tunneling mechanism (<326.3 kV/cm) and space-charge-limited conduction (SCLC) (log J∝2log E) were involved in the leakage behavior of the BFO_N_ film. From Figure 5d,f, the SCLC mechanism (<100.4 kV/cm) and the FN tunneling mechanism (>100.4 kV/cm) were predominant under a positive electric field. The Ohmic conduction mechanism (<150.6 kV/cm) and FN tunneling mechanism (>150.6 kV/cm) can be observed under the negative electric field.

**Figure 6:**
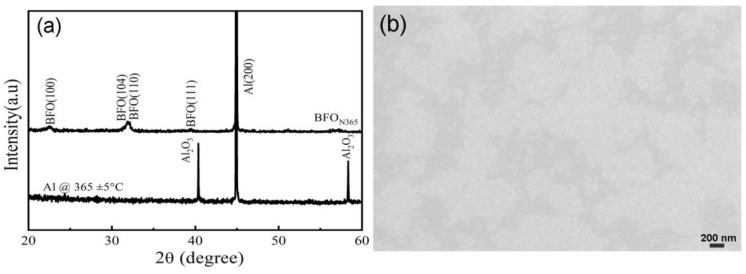
XRD 2θ-scan patterns (**a**) of the BFO_N365_ film and Al foil annealed at 365 ± 5 °C and the surface SEM image (**b**) of the BFO_N365_ film.

**Figure 7:**
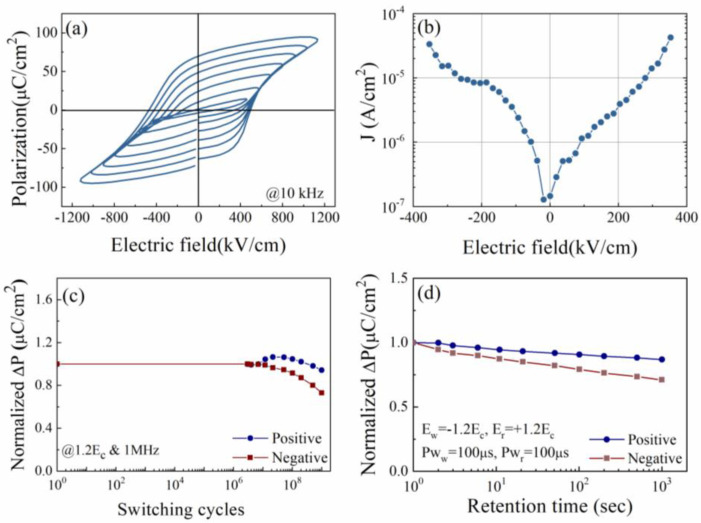
(Color online) room-temperature (**a**) P–E curves and (**b**) leakage property, and the normalized pulsed polarization as a function of (**c**) switching cycles and (**d**) the retention time for the BFO_N365_ film.

According to the above discussion, it can be concluded that by adopting a N_2_-rich atmosphere, improved crystalline quality could be achieved in the BFO films at 440 ± 5 °C, resulting in better ferroelectric properties. Furthermore, a lower annealing temperature was expected for the BiFeO_3_ films with Al substrates and a N_2_-rich atmosphere. Therefore, an attempt was made to fabricate the BFO films below the annealing temperature of 400 °C. Finally, BFO films with adequate electric properties were achieved a low temperature of 365 ± 5 °C (BFO_N365_) on Al substrates under a N_2_-rich atmosphere. The detailed properties of the BFO_N365_ film are shown in Figure 6 and Figure 7.

### 3.2. Performance of BiFeO_3_ Films Annealed at 365 ± 5 °C under a N_2_-Rich Atmospheres 

#### 3.2.1. Microstructure Analysis 

The XRD 2θ-scan pattern and surface SEM image of the BFO_N365_ film are shown in Figure 6. A bulk-like random polycrystalline structure with obvious (100) and (110)/(104) peaks was observed, indicating that the BFO films were crystallized even at a low annealing temperature of 365 ± 5 °C. A dense morphology with fine nanograins was clearly revealed, indicating that the film had crystallized at this low annealing temperature.

#### 3.2.2. Room-Temperature Electrical Properties

Typical P-E loops were observed in Figure 7a for the BFO_N365_ film, and a sizable remnant polarization (P_r_~70 μC/cm^2^) and good rectangularity (~75.0%) of the hysteresis loop at the applied electric field of 1118.1 kV/cm were obtained. These observations indicate that Al foil serves as a suitable substrate for fabricating ferroelectric BiFeO_3_ films under low annealing temperatures. As shown in Figure 7b, the leakage current density of BFO_N365_ was lower than ~1 × 10^−4^ A/cm^2^ under the electric field of 353.5 kV/cm. Figure 7c,d illustrates that good fatigue resistance with a low loss (~5.8%) of up to 1.0 × 10^9^ switching cycles for positive polarization was observed, and the loss for negative polarization under the same testing conditions was ~27%. Retention testing was carried out for BFO_N365_ films; a degradation of ~29% for the negative polarization was observed after 1.0 × 10^3^ s, which is much higher than that for the positive polarization (~14%). The improved fatigue and retention performance at the positive side could be ascribed to the reduced interfacial defects (e.g., vacancies, grain boundaries or amorphous regions) at the bottom electrode after a long exposure to a processing temperature. The achieved ferroelectric characteristics of a BFO film directly deposited onto the substrate without a buffer layer under the temperature of 400 °C have not previously been reported. This was attributed to the N_2_-rich atmosphere, the Al foil substrate, as well as the homogeneous precursor solution.

## 4. Conclusions

In summary, BFO films were successfully fabricated at a low annealing temperature (<450 °C). A common base metal, aluminum foil, was adopted as the bottom electrode and substrate for the BFO ferroelectric film. Compared with an oxygen-rich atmosphere, a nitrogen-rich atmosphere was more conducive to optimizing the performance of BFO films. Along with excellent retention and fatigue properties, *P-E* loops with a large *P_r_* value (~78.1 μC/cm^2^) and high rectangularity (~91.3%) at the applied electric field of 1165.2 kV/cm were obtained in the BFO films deposited under a N_2_-rich atmosphere at 440 ± 5 °C. By using a N_2_-rich atmosphere as well as Al substrates, BFO films with good electrical properties were achieved a very low temperature of 365 ± 5 °C. This offers a new strategy for lowering the annealing temperature of BFO films. 

## Figures and Tables

**Table 1:** The average grain size for the BFO_N_ and BFO_O_ films.

Film		BFO_N_	BFO_O_
Global average grain size via the Scherrer formula	(100)-oriented grains	180 nm	120 nm
	(111)-oriented grains	75 nm	50 nm
	(211)-oriented grains	30 nm	23 nm
Local average grain size via SEM analysis (using 100 grains)		105 nm	55 nm

## Data Availability

Data are available upon request to the corresponding author.
